# The Role of Thermogenic Fat Tissue in Energy Consumption

**DOI:** 10.3390/cimb44070219

**Published:** 2022-07-11

**Authors:** Masato Horino, Kenji Ikeda, Tetsuya Yamada

**Affiliations:** Department of Molecular Endocrinology and Metabolism, Tokyo Medical and Dental University, Tokyo 113-8519, Japan; masamem@tmd.ac.jp (M.H.); tyammem@tmd.ac.jp (T.Y.)

**Keywords:** non-shivering thermogenesis, thermogenic fat tissue, uncoupling protein 1, beige adipocytes, brown adipocytes, glucose metabolism

## Abstract

Mammalian adipose tissues are broadly divided into white adipose tissue (WAT) and thermogenic fat tissue (brown adipose tissue and beige adipose tissue). Uncoupling protein 1 (UCP1) is the central protein in thermogenesis, and cells that exhibit induced UCP1 expression and appear scattered throughout WAT are called beige adipocytes, and their induction in WAT is referred to as “beiging”. Beige adipocytes can differentiate from preadipocytes or convert from mature adipocytes. UCP1 was thought to contribute to non-shivering thermogenesis; however, recent studies demonstrated the presence of UCP1-independent thermogenic mechanisms. There is evidence that thermogenic fat tissue contributes to systemic energy expenditure even in human beings. This review discusses the roles that thermogenic fat tissue plays in energy consumption and offers insight into the possibility and challenges associated with its application in the treatment of obesity and type 2 diabetes.

## 1. Introduction (What Is Thermogenic Fat Tissue?)

Mammalian adipose tissues are broadly divided into two types: white adipose tissues (WAT) and thermogenic fat tissue, the latter of which consists of brown adipose tissue (BAT) and beige adipose tissue. WAT comprises white adipocytes that possess a large unilocular lipid droplet, and its role is to store excess energy in the body in the form of neutral fat (triglyceride) [1]. By contrast, BAT comprises brown adipocytes that possess multilocular lipid droplets and abundant mitochondria and function as thermogenic adipocytes that consume energy [1]. Beige adipocytes are induced scatteredly throughout WAT by various stimuli, and this phenomenon is referred to as “beiging”. Long-term cold stimulation, sympathetic nerve stimulation, peroxisome proliferator-activated receptor γ (PPARγ) agonists, and exercise reportedly induce beiging [2,3,4].

Uncoupling protein 1 (UCP1), which is expressed in the inner mitochondrial membrane, plays a pivotal role in the thermogenic mechanism of brown adipocytes. UCP1 is activated through stimulation of the sympathetic nervous system, dissipates the proton gradient generated by the electron-transport chain, and releases energy as heat [5]. This thermogenic mechanism is referred to as non-shivering thermogenesis.

In human beings, brown adipocytes were previously detected only in neonates, resulting in the assumption that they undergo atrophy or have no physiological significance in adulthood [6]. However, with the advent of ^18^F-fluorodeoxyglucose (FDG)-positron emission tomography (PET)/computed tomography (CT), it was revealed that human adults also possess physiologically active brown adipocytes in the neck region [7,8]. Through RNA sequencing (RNA-seq), it was shown that human brown adipocytes are similar to mouse beige adipocytes rather than classical mouse brown adipocytes [9,10], suggesting that human beings possess an inducible form of thermogenic adipocytes that contribute to energy consumption.

Obesity is defined as excessive fat accumulation and is diagnosed at a body mass index ≥30 kg/m^2^ [11]. Obesity increases the incidence of type 2 diabetes, which is a worldwide concern based on its correlation with fatal cardiovascular diseases or microvascular complications, such as neuropathy, retinopathy, and nephropathy. Currently, nearly 400 million people have type 2 diabetes, and the number is expected to increase to ~600 million by 2040 [12]. Type 2 diabetes is characterized by insulin resistance and/or deficiency caused by pancreatic β cell function. Several studies reported that thermogenic fat tissue positively correlates with improved insulin sensitivity in human beings [2,8,13,14]. This finding suggests that thermogenic fat tissue might play a role in the treatment of obesity and type 2 diabetes, although there are many limitations to its actual application for treatment in human beings.

This review discusses the roles that thermogenic fat tissue plays in energy consumption and offers insight into the challenges associated with its application in the treatment of obesity and type 2 diabetes.

## 2. Origin and Regulators of Thermogenic Adipocytes

Classical brown adipocytes differentiate from muscle progenitor cells expressing myogenic factor 5 (MYF5). Progenitors expressing paired box 7 (PAX7) and engrailed-1 also differentiate into either brown adipocytes or muscle cells. A previous study reported that these progenitors occur at embryonic days 9.5 to 11.5 in mouse embryos [15]. While cells expressing myoblast determination protein 1 (MYOD), the downstream of MYF5, promote the differentiation of skeletal muscle cells, MYOD-negative cells develop into brown adipocytes. Early B-cell factor 2 (EBF2) is identified as a specific gene related to embryonic brown progenitors [16].

**Figure 1:**
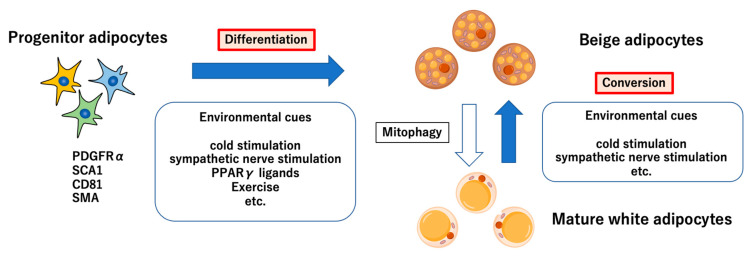
The origins of beige adipocytes. There are two mechanisms. One is that differentiate to beige adipocytes from progenitor adipocytes by environmental cues such as cold stimulation, sympathetic nerve stimulation, PPARγ ligands and exercise. On the other hand, conversion from mature white adipocyte to beige adipocyte also might exist under cold stimulation and sympathetic nerve stimulation. These recruited beige adipocytes return to white fat signatures by mitophagy following the withdrawal of environmental cues.

Beige adipocytes differentiate from MYF5-negative cells. There are two hypotheses on the origin of beige adipocytes (Figure 1). One is that beige adipocytes newly differentiate from preadipocytes expressing platelet-derived growth factor receptor α (PDGFRα), and the other is that they convert from mature adipocytes. An in vivo study in AdipoChaser mice reported that many of the beige adipocytes induced by cold stimulation or β agonists are not derived from existing mature adipocytes [17], and recently, Pdgfra^+^ Sca1^+^ Cd81^+^ cells have been identified as beige progenitor adipocytes [18]. Moreover, a report suggests that 50% to 70% of beige adipocytes in mice reared under cold stimulation for 1 week originated from smooth muscle actin (SMA)-positive progenitor cells [19]. SMA is expressed in smooth muscle cells. Given that beige-adipocyte formation around capillaries can be facilitated by retinoic acid [20], this in vitro finding suggests that perivascular smooth muscle cells are also candidates for the origin of beige adipocytes.

On the other hand, an in vitro study reported that mature adipocytes can undergo conversion to beige adipocytes [21]. Cold exposure and β_3_-adrenergic receptor agonists are said to be the external stimuli, and when mice were exposed to 6 °C, approximately 50% of beige adipocytes were derived from existing adipocytes [22]. It is said that after the withdrawal of external stimuli, these recruited beige adipocytes return to white fat signatures such as unilocular lipid droplets and low mitochondria [22]. This is due to mitophagy, a form of autophagy for mitochondrial degradation. A recent report shows that the blockade of mitophagy prevents recruited beige adipocytes from returning to white adipocytes even after the external stimuli end [10].

A recent study using single-cell RNA-seq of adipose tissue revealed dipeptidyl peptidase 4 (DPP4) as an adipose stem cell marker [23]. DPP4-positive cells are proliferative and multipotent and have the capacity to differentiate into preadipocytes. This hierarchy in adipocyte differentiation might also exist in beige adipocytes, though it remains to be proven. The niche (the microenvironment of a specific tissue) might also be important for the development of beige adipocytes. The adipose tissue niche comprises immune cells, fibroblasts, and endothelial cells [24], and their interaction with preadipocytes might be necessary for the in vivo development of beige adipocytes.

A well-known regulatory factor of beige-adipocyte differentiation is the transcription factor PR domain-containing 16 (PRDM16), which was previously known as a factor that regulates brown-adipocyte differentiation [25]. PRDM16 binds to PPARγ coactivator 1 alpha (PGC1α) and CCAAT/enhancer-binding protein beta (C/EBP-β) and is involved in the induction of beige-adipocyte differentiation. Another transcription factor, EBF2, also functions in the differentiation of both brown and beige adipocytes [26]. Moreover, cell death-inducing DFFA-like effector A (CIDEA) is reported to translocate from the cytoplasm to the nucleus and inhibit liver X receptor expression, contributing to UCP1 expression in white adipocytes [27]. Thus, these reports suggest that factors important for brown-adipocyte differentiation also play a functional role in beige-adipocyte differentiation. However, the regulatory mechanisms underlying the differentiation of these two types of cells are not the same. For example, transducin-like enhancer of split 3, which is abundant in WAT, suppresses CIDEA through the inhibition of EBF2 following the disappearance of cold stimulation [28]. Furthermore, a recent report showed that PPARγ and PPARα function cooperatively in beiging [29]. Future work is needed to provide insight for elucidating the regulatory mechanisms of differentiation specific to beige adipocytes.

## 3. The Role of UCP1-Dependent Thermogenesis

In the late 1970s, a study demonstrated that cell respiration (internal respiration) in mitochondria is the main determinant of non-shivering thermogenesis [30]. Subsequently, in the 1980s, the molecular mechanisms of UCP1 expression in the mitochondrial inner membrane were elucidated [31]. From the late 20th century to the early 21st century, successive reports indicated that Ucp1-knockout mice cannot maintain body temperature under cold conditions [32,33], leading to the notion that non-shivering thermogenesis is UCP1-dependent.

UCP1-dependent thermogenesis occurs when the sympathetic nervous system is activated, which in turn results in norepinephrine secretion [34]. Norepinephrine binds to the β adrenergic receptor on the adipocyte membrane. Specifically, the signal to the β_3_-adrenergic receptor activates adenylate cyclase, resulting in elevation of cyclic adenosine monophosphate levels and activation of protein kinase A (PKA) [34]. PKA activates UCP1 and the breakdown of triglycerides into fatty acid and glycerol, which are used as substrates for thermogenesis [34,35].

Several studies reported an association between UCP1-dependent thermogenesis and glucose metabolism. BAT-deficient mice created by transgenic expression of diphtheria toxin under Ucp1-promoter regulation showed obese and diabetic phenotypes [36]. Additionally, in an experiment in which brown adipocytes in mice were transplanted into visceral fat tissues of other mice, increased glucose uptake by the fat tissues of the recipients improved blood glucose levels during glucose-tolerance tests, and improved insulin sensitivity according to insulin-tolerance tests was observed 8 to 12 weeks after transplantation [37]. Moreover, the fact that UCP1 expression was maintained in the transplanted brown adipocytes suggested the presence of UCP1-dependent thermogenic mechanisms [37]. Furthermore, in vitro experiments showed that oxygen consumption in brown adipocytes is dependent on glucose and fatty acid metabolism, and that knockdown of glucose transporters (GLUTs) decreases oxygen consumption in brown adipocytes [38]. Recently, another study reported that UCP1 overexpression in human white adipocytes increased glucose uptake, whereas GLUT1 inhibition impaired this effect on glucose uptake [39]. Therefore, these findings suggest that UCP1-dependent thermogenic mechanisms at least partially involve the facilitation of glucose uptake.

**Figure 2:**
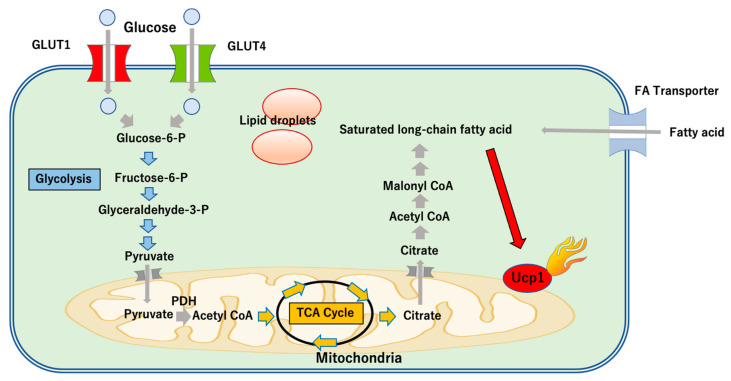
Glucose uptake in beige adipocytes. Glucose uptake by GLUT1 or GLUT4 is used for glycolysis via glucose-6-phosphate, and pyruvate subsequently moves into mitochondria. Citrate is generated under the tricarboxylic acid cycle, and changes to acetyl CoA after moving into the cytoplasm, where fatty acids are finally made. Fatty acids also derive from outside the adipocytes. Long-chain fatty acids are the main substance in UCP1-dependent thermogenic mechanism. Glucose-6-P: glucose-6-phosphate, Fructose-6-P: fructose-6-phosphate, Glyceraldehyde-3-P: glyceraldehyde-3-phosphate, TCA: tricarboxylic acid, FA: fatty acid, PDH: pyruvate dehydrogenase.

Glucose is taken up into the cells through GLUT1 or GLUT4. This glucose is used for glycolysis via glucose-6-phosphate, and pyruvate subsequently moves into mitochondria (Figure 2). Citrate is generated under the tricarboxylic acid cycle, and changes to acetyl CoA after moving into the cytoplasm, where fatty acids are finally made. Fatty acid is the main substance in UCP1-dependent thermogenic mechanisms [40], with recent reports indicating that the long-chain fatty acids activate UCP1 [41,42]. It is conceivable that fatty acid produced by lipolysis also plays an important role under cold exposure when brown and beige adipocytes produce heat. Interestingly, a recent study in mice suggested that lipolysis in white adipocytes due to cold stimulation and β_3_ adrenergic receptor stimulates insulin secretion and contributes to the facilitation of lipid uptake into activated brown adipocytes [43]. Of note, disruption of insulin signaling impairs glucose uptake, lipid uptake, and thermogenesis in brown adipocytes [43]. This study suggests that insulin signaling regulates substrates for thermogenesis. Further studies will be needed to understand how thermogenesis and glucose homeostasis are regulated in type 2 diabetes.

## 4. The Role of UCP1-Independent Thermogenesis

Until recently, only UCP1 was thought to contribute to non-shivering thermogenesis; however, UCP1-independent thermogenic mechanisms have subsequently been demonstrated. A report suggested that Ucp1-knockout F1 mice created by crossing C57BL/6J mice with 129/SvlmJ mice were able to maintain body temperature under cold stimulation [44]. Additionally, *Ucp1*-knockout mice created using pure C57BL/6J mice can also maintain body temperature under long-term cold stimulation [45]. Therefore, the presence of UCP1-independent thermogenic mechanisms has been described, at least in mice.

**Figure 3:**
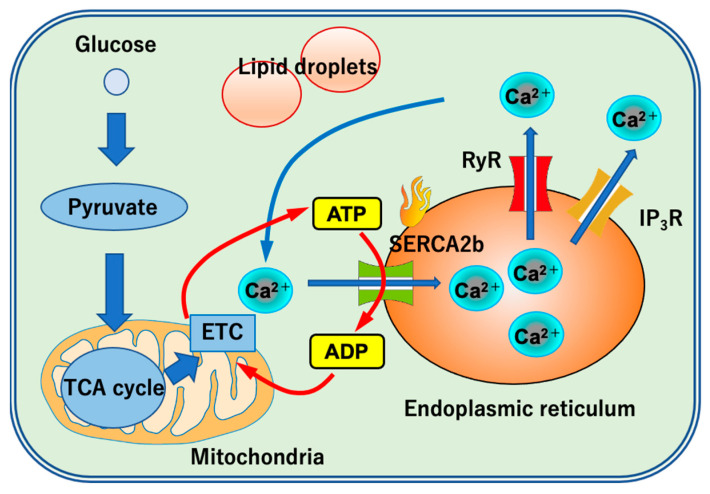
Ca^2+^ cycling in beige adipocytes. Ca^2+^ flows into the cytoplasm through RyR and IP_3_R. The Ca^2+^ uptake into the endoplasmic reticulum again by SERCA2b leads to usage of ATP, which is one of the UCP1-independent thermogenic mechanism. SERCA: Sarco-endoplasmic reticulum ATPase, RyR: ryanodine receptor, IP_3_R: Inositol 1,4,5-triphosphate receptor, TCA: tricarboxylic acid, ETC: electron-transport chain.

One UCP1-independent thermogenic mechanism is thought to involve Ca^2+^ cycling mediated by sarco-endoplasmic reticulum ATPase (SERCA) (Figure 3). The endoplasmic reticulum is the main storage site of Ca^2+^ in the cell. Ca^2+^ flows into the cytoplasm through the ryanodine receptor (RyR) and inositol 1,4,5-triphosphate receptor (IP_3_R) located on the endoplasmic reticulum membrane. Subsequently, Ca^2+^ uptake into the endoplasmic reticulum again by SERCA results in production of adenosine diphosphate (ADP). Skeletal muscles are reported to have the ability of non-shivering thermogenesis by Ca^2+^ cycling [46], and an in vitro study previously reported the involvement of this process in non-shivering thermogenesis in mouse brown adipocytes [47]. Recently, an in vivo study reported the same findings based on a mouse model created by crossing adipocyte-specific PRDM16-overexpression mice, using aP2 (fatty acid-binding protein 4) promoter, and Ucp1-deficient mice (i.e., mice that do not express UCP1, despite an increased number of beige adipocytes), reported a significant increase in UCP1-independent thermogenesis and glucose uptake in subcutaneous adipose tissue as compared with those in control Ucp1-deficient mice [48]. Furthermore, elevated levels of Ryr2 and Serca2b expression in the subcutaneous adipose tissue of these genetically modified mice were demonstrated [48], suggesting the importance of a UCP1-independent thermogenic mechanism mediated by Ca^2+^ cycling, particularly in thermogenesis in beige adipocytes. SERCA activity contributes to thermogenesis [49], so SERCA activity regulate molecules could be possible targets for clinical application of UCP1-independent thermogenesis.

Sarcolipin (SLN) is potentially involved in Ca^2+^ cycling and binds to SERCA to facilitate ATP hydrolysis. In contrast to mice capable of maintaining body temperature under cold stimulation following the removal of brown adipocytes, *Sln*-deficient mice were unable to maintain body temperature when brown adipocytes were removed [50]. Based on another report showing that *Sln*-deficient mice become obese when fed a high-fat diet [51], these findings suggest that SLN is involved in UCP1-independent thermogenic mechanisms and can potentially enhance energy consumption. Of note, SLN expression is quite low in beige adipocytes [48]. Future studies need to identify Ca^2+^ cycling regulators in beige adipocytes.

Creatine cycling represents a different type of UCP1-independent thermogenic mechanism. Creatine is involved in energy production, particularly in muscles, during rapid movement. Specifically, phosphocreatine reacts with ADP to synthesize creatine and ATP. A previous study showed that internal respiration in mitochondria mediated by creatine cycling was facilitated in the beige adipocytes of mice reared under cold stimulation [52]. Additionally, mice with an adipocyte-specific deficiency of glycine amidinotransferase, which is involved in creatine biosynthesis, are unable to maintain body temperature under cold stimulation, despite the presence of UCP1 expression in brown adipocytes [53]. It is also suggested that expression of creatine kinase B (CKB), which localizes to the mitochondria of brown adipocytes, increases under cold stimulation, and that CKB-deficient mice showed a significant increase in body weight and elevation in fasting blood glucose level [53]. Further research on the role of CKB in human beings is necessary to determine its potential as a therapeutic target for type 2 diabetes.

The influence of proton (H^+^) outflow from mitochondria is considered another UCP1-independent thermogenic mechanism and was suggested by the observation that increased H^+^ outflow increases oxygen consumption by mitochondria [54]. It is conceivable that an ADP/ATP carrier (AAC) in the mitochondrial inner membrane is a key player in this phenomenon, with a recent study reporting AAC-mediated H^+^ outflow in UCP1-deficient mitochondria [55].

Another study reported beiging in subcutaneous adipose tissue of *Ucp1*-knockout mice with enhanced function of AMP-activated protein kinase (AMPK), which is involved in energy production [56]. Interestingly, feeding these mice a high-fat diet induced the expression of skeletal muscle-associated genes in subcutaneous adipose tissue, suggesting that AMPK contributes to the increased oxygen consumption in subcutaneous adipose tissue. The elucidation of UCP1-independent thermogenic mechanisms might be important particularly for the treatment of obese patients.

## 5. Factors Inducing Thermogenic Adipocytes

In parallel with the elucidation of both UCP1-dependent and -independent mechanisms for energy consumption, many factors have been reported that induce thermogenic adipocytes. PPARγ agonists (specifically rosiglitazone) induce beige adipocytes. Rosiglitazone stabilizes PRDM16 expression [57], and an in vitro study reported that the beiging function of rosiglitazone is enhanced in the presence of β_3_-adrenergic receptor agonists [58], suggesting that there is at least partial overlap between this mechanism and beiging induced by long-term cold stimulation or sympathetic nerve stimulation. Interestingly, this study also reported increased expression of β_3_-adrenergic receptor following administration of PPARγ agonists during the first three days rather than across the entire period of adipose-tissue differentiation (seven days) [58], indicating that the beiging effect of PPARγ agonists is limited to a certain period.

Fibroblast growth factor 21 (FGF21) also induces beige adipocytes. Although FGF21 is a hormone secreted by the liver during hunger, it is also secreted by brown and beige adipocytes under cold stimulation or β adrenergic receptor stimulation, which induces UCP1 expression via activation of PGC1α [59]. FGF21 was found to stimulate glucose uptake through the activation of GLUT1 in mice [60]. Glucose taken up via GLUT1 is used in the synthesis of fatty acids, which are the main substrates involved in UCP1-dependent thermogenesis [40], implying that FGF21-related beiging contributes to glycemic control.

Irisin, a myokine secreted by skeletal muscles, reportedly induced beige adipocytes during exercise. Irisin acts on white adipocytes and increases UCP1 expression through p38 mitogen-activated protein kinase and extracellular-signal regulated kinase signaling [61]. GLUT4 induction is possibly involved in irisin-related beiging [62].

The cytokine interleukin-6 (IL-6) is also secreted by skeletal muscles during exercise, with a recent study showing increased IL-6 secretion from beige adipocytes rather than white adipocytes in human beings [63]. This study demonstrated that continuous blocking of the IL-6 receptor during beige differentiation resulted in morphological changes characteristic of white adipocytes [63]. Another report suggested the involvement of signal transducer and activator of transcription 3 (STAT3) in IL-6 regulation of beiging of white adipocytes by enhancing PPARγ and *UCP1* transcription [64]. Given that *Il6*-knockout mice show decreases in STAT3 activity and the beiging potential of white adipocytes, this suggests that IL-6/STAT3 signaling might be a target for beiging.

Group 2 innate lymphoid cells (ILC2s), which work in response to tissue damage and allergen exposure, also play a potential role in beige-adipocyte biogenesis. IL-33 reportedly stimulates ILC2s, resulting in the secretion of IL-5 and IL-13 [65], which promote the proliferation of adipocyte precursors and the biogenesis of beige adipocytes. Although the IL-33 level is upregulated in obese adipose tissue, this does not coincide with upregulated beiging [66]. Adipocytes are reportedly the main source of cold-induced IL-33 secretion [67], suggesting a possible dysfunction in IL-33 response in obese adipose tissue. A recent study reported chemerin, a newly identified adipokine, and its receptor, chemerin chemokine-like receptor 1, as regulators of IL-33-induced beige-adipocyte biogenesis [67]. Further research is needed to determine the effectiveness of ILC2-related beiging in glucose metabolism.

Bone morphogenetic proteins (BMPs) may also play a role in inducing thermogenic adipocytes. *Bmp7*-knockout embryos do not express UCP1, and BMP7 reportedly promotes the beiging of WAT by increasing PGC1α expression [68]. Another study showed that BMP8b enhances the intracellular response to adrenergic stimulation, resulting in the thermogenesis of mature brown adipocytes [69]. Additionally, a possible role for BMP9 in energy consumption is particularly interesting, given its role in enhancing FGF21 expression in animal experiments [70]. Furthermore, patients with type 2 diabetes reportedly show lower circulating BMP9 levels relative to healthy subjects [70]. Therefore, upregulation of BMP9 might be a promising strategy for glycemic control in human beings, although this remains to be confirmed.

## 6. Clinical Relevance of Thermogenic Adipose Tissue in Human Beings

The average mass of BAT is 50–70 g [71]. BAT is said to contribute between 2% and 5% of the resting metabolic rate in human beings [72,73]. A study evaluating BAT oxygen consumption showed that BAT consumed approximately 15–25 kcal/day under mild cold conditions (15.5 °C) [74]. Another study used radiological 3D mapping to estimate that BAT contribution is 27–123 kcal/day at room temperature, and this level increases to 46–211 kcal/day under cold conditions [75,76].

Several studies have investigated the changes in glucose uptake activity in human BAT triggered by exposure to cold conditions. When young healthy individuals were exposed to a room temperature of 17 °C for 2 h per day over a period of six weeks, significant reduction in total fat mass, which correlated with glucose uptake in BAT, was observed [8]. Another study that investigated longer periods of exposure to cold conditions in which healthy individuals slept in a temperature-controlled unit at night for four months, revealed that cold temperatures triggered an increase in insulin sensitivity and BAT activity [2]. Similar effects were also observed in patients with type 2 diabetes. After exposure to room temperatures between 14–15 °C for 2 h each day over a period of 10 days, patients with type 2 diabetes had increased insulin sensitivity, which was associated with an increase in glucose uptake in BAT [14]. The activation of BAT and increase in energy consumption were also observed in obese individuals [77], who were said to have less BAT than non-obese individuals [78].

Studies have also shown the effect of cold-induced BAT activation on glucose metabolism. A study conducted in the U.S. showed that cold-induced BAT activation increased the clearance of plasma glucose [79]. A cross-sectional analysis conducted in China also showed that individuals with active BAT had lower fasting insulin and insulin resistance than individuals with inactive BAT [80]. In addition, a study conducted in Japan revealed that the impact of cold-activated BAT on hemoglobin A1c (HbA1c) was independent of body fat [81]. These findings indicate that BAT activation can be used to improve glucose metabolism.

The next step is to determine the mechanism of BAT activation in human beings, since oral or intravenous medication would be easier for clinical use than exposure to cold conditions. However, the clinical use of most substances that activate thermogenic adipose tissue is controversial. The effect of FGF21 and its analogs on glucose metabolism has been investigated, but a recent report suggested that beiging was not influenced by the concentration of FGF21 circulating in the blood [82]. Moreover, in clinical trials, FGF21 analogs did not show adequate glucose-lowering effects [83], so it remains unclear whether the beiging effect of FGF21 is related to glycemic control. Further research is needed to examine the application of FGF21 in the treatment of type 2 diabetes.

Irisin is reported to stimulate beiging in specific types of human adipocytes [84], and a positive association between circulating irisin levels and body weight was also reported [85], but the effect of irisin on type 2 diabetes has not yet been established. Furthermore, accurate and sensitive assays need to be developed as the concentration of irisin in human serum/plasma is 5–278-fold lower than the detection limit of ELISA kits, which makes it difficult to accurately measure the irisin concentration during energy consumption in human beings [86].

Although IL-6 reportedly contributes to beiging [63], a recent review indicated that an increase in IL-6 levels delays gastric emptying and consequently decreases the postprandial glucose concentration in human plasma [87]. Therefore, the relationship between IL-6-mediated beiging and glucose metabolism also requires investigation.

Another theory is that food intake increases the body’s total energy consumption, a phenomenon known as diet-induced thermogenesis; however, this was disproved by a recent study that did not observe an association between BAT activity and calorie intake in 102 human adults [88]. Research on the use of dietary supplements to increase energy consumption has also been conducted. Capsaicin and capsinoids, the non-pungent analogs of capsaicin, were reported to induce BAT activation and reduce body fat in human beings [89,90,91,92].

## 7. Current Therapeutic Drugs Capable of Increasing Energy Consumption in Human Beings

There are currently no drugs capable of inducing beiging available for the treatment of type 2 diabetes or obesity. Currently, PPARγ agonists, specifically thiazolidine drugs represented by pioglitazone, are used for the treatment of type 2 diabetes. These drugs improve insulin resistance through two pathways. One is PPARγ activation strongly facilitating fat accumulation in WAT, resulting in improved levels of ectopic fat in skeletal muscles and the liver under obese conditions. The other involves decreased secretion of tumor necrosis factor-α and other inflammatory cytokines, the levels of which are elevated during obesity, resulting in the facilitation of adiponectin secretion. Although the administration of pioglitazone to human adipocytes facilitates beiging in vitro, to the best of our knowledge, there are no reports that oral administration of pioglitazone facilitates glucose uptake by human brown adipocytes. By contrast, a study reported decreased brown-adipocyte activity under cold stimulation in a group administered pioglitazone [93]. Therefore, the clinical usefulness of beiging induced by thiazolidine drugs is still under debate.

With respect to β_3_-adrenergic receptor stimulation, which strongly induces beige adipocytes, numerous studies have evaluated the use of mirabegron. One report from a study of women in their 20s with no underlying disease showed that long-term administration of mirabegron (4 weeks) increased the metabolic activity of brown adipocytes [94]. Another study in obese patients with insulin resistance showed that taking mirabegron facilitated the breakdown of fat in subcutaneous adipose tissue, improved blood glucose levels according to glucose-tolerance tests, and improved HbA1c values [95]. It is conceivable that mirabegron, the application of which is currently limited to the treatment of overactive bladder, may be useful as a therapeutic drug for type 2 diabetes or obesity.

In terms of associations with FGF21, sodium-glucose transport protein-2 (SGLT-2) inhibitor is being used for the treatment of type 2 diabetes, with the associated mechanisms involving inhibition of glucose absorption in renal tubules. Part of this mechanism also involves hypoglycemic effects that facilitate the breakdown of fat and reduce adipose tissue. The possible involvement of FGF21 in these activities was reported in a study using a mouse model [96]. Recently, another report indicated that 8-week administration of empagliflozin to mice fed a high-fat diet reduced their body weight and induced beige adipocytes [97]. Therefore, it is conceivable that the improvements observed in association with SGLT-2 inhibitors on glucose metabolism may involve beiging.

Additionally, other reports suggest an association between glucagon-like peptide 1 agonists and beiging. Injection of liraglutide into rats fed a high-fat diet for 12 weeks induced the expression of markers of beige adipocytes in subcutaneous adipose tissue [98]. Moreover, transfection experiments with 3T3-L1 adipocytes showed that microRNA-27b attenuated the effect of liraglutide, which showed an increase in the levels of beiging markers such as UCP1 and PRDM16 [99].

It is also important to consider the side effects of these drugs. Specifically, PPARγ agonists can cause fluid retention, and their use is not recommended in patients who have concomitant heart failure. Additionally, in the case of mirabegron, its use should be limited for patients with urinary retention and hypertension, and risks associated with dehydration and urinary tract infection need to be considered for SGLT-2 inhibitors. Future studies are required to identify drugs with higher efficacy and lower risks of adverse side effects.

## 8. Discussion

In this review, we discussed the role of thermogenic adipose tissue in increasing energy consumption and the factors potentially involved. Among the factors mentioned, the most applicable for clinical usage is β_3_-adrenergic receptor stimulation, with evidence that it improves blood glucose levels in human beings [95]. The fact that the dosage of mirabegron used in this study was FDA-approved and the detection of BAT activity was done by ^18^FDG-PET/CT also supports its feasibility for clinical application. It has been shown by more than 130,000 scans from nearly 50,000 patients that BAT activity detected by ^18^FDG-PET/CT is independently correlated with lower incidence of type 2 diabetes [100]. However, there are risks associated with excess radiation exposure, and alternative methods are currently being researched. A recent study showed that magnetic resonance imaging (MRI) can also detect BAT activity [101], but since the current MRI assessment comprises both active and inactive states of BAT [102], we think that ^18^FDG-PET/CT remains the gold standard to reflect BAT activity.

This review has limitations. First, many of the results are based on animal models. Regarding in vivo human studies, some reports showed a correlation between BAT activation and increased glucose uptake [2,14]; however, the mechanism involved in this relationship has not been elucidated. Given the existence of both UCP1-dependent and -independent thermogenesis, it is important to investigate the relative contribution of these two mechanisms to energy consumption in human beings. Second, it remains difficult to accurately measure the BAT content in human beings. Although the development of ^18^FDG-PET/CT has accelerated BAT studies in human beings, this imaging tool reportedly underestimates the actual content of BAT; the influence of BAT on energy consumption may therefore be larger than that reported [103]. Moreover, in the case of human WAT, a previous report demonstrated the difficulty in determining the effectiveness of beiging agents. The previous report estimated that, to achieve 1% of the UCP1 content in BAT necessary to increase energy consumption in human beings, a 10-fold increase in UCP1 expression would be required [104]. There are no clinically available methods to estimate UCP1 levels in human fat, and—given the possible existence of UCP1-independent thermogenesis—measuring UCP1 alone is unlikely to reflect the actual energy consumption. For therapeutic applications in the treatment of type 2 diabetes and obesity, it is necessary to discover a molecule that allows accurate measurement of BAT levels in human serum/plasma to promote future studies into the contribution of thermogenic adipose tissue to energy consumption.

## 9. Conclusions

Among the factors that have been previously reported to induce thermogenesis, β_3_-adrenergic receptor stimulation currently has the highest potential for clinical usage because of evidence from human studies showing that activation of thermogenic adipose tissue leads to energy consumption. As a measurement, ^18^FDG-PET/CT is the best method to reflect the effect of energy consumption of thermogenesis fat. ^18^FDG-PET/CT is not perfect in terms of radiation exposure and underestimation, so it is necessary to identify other therapeutically applicable molecules. Recent studies showed that beige adipocytes are diverse in origin and thermogenic mechanisms, including UCP1-independent mechanisms. Future studies of this diversity in human beings may provide insights to improve clinical approaches.
